# Extraskeletal Myxoid Chondrosarcoma of the Vulva: A Case Report

**DOI:** 10.7759/cureus.33601

**Published:** 2023-01-10

**Authors:** Maya Omote, Hiroshi Tsubamoto, Yoshihiro Ide, Kenichiro Kawai, Hiroyuki Futani, Hiroaki Shibahara

**Affiliations:** 1 Department of Obstetrics and Gynecology, School of Medicine, Hyogo Medical University, Nishinomiya, JPN; 2 Department of Pathology, School of Medicine, Hyogo Medical University, Nishinomiya, JPN; 3 Department of Plastic Surgery, School of Medicine, Hyogo Medical University, Nishinomiya, JPN; 4 Department of Orthopaedic Surgery, School of Medicine, Hyogo Medical University, Nishinomiya, JPN; 5 Department of Obstetrics and Gynecology, School of Medicine, Hyogo Medical University, Hyogo, JPN

**Keywords:** vulva, pazopanib, radiation therapy, extraskeletal myxoid chondrosarcoma, case report

## Abstract

Extraskeletal myxoid chondrosarcoma (EMC) of the vulva is extremely rare. We report our experience with a case of disease control by radiation therapy to a localized lesion of EMC. A 41-year-old woman presented to our clinic with a vulvar mass. Magnetic resonance imaging showed a 15 cm mass between the perineum and the medial thigh muscle. It was the "adductor magnus muscle." After the needle biopsy, a histopathological diagnosis of EMC was made. Tissue genomic analysis detected the EWSR1-NR4A3 fusion gene. A joint operation by the Department of Orthopedics, Gynecology, and Plastic Surgery was performed, which included a wide excision of the perineum, partial excision of the medial thigh muscle, and rectus abdominis valvuloplasty.

Intraoperatively, pubic infiltration was detected. Postoperative pelvic radiotherapy was administered as adjuvant therapy. Recurrent common iliac lymph node metastases outside the irradiation field and multiple lung metastases were observed. Pazopanib was administered as adjuvant therapy. Pulmonary metastases were controlled, but the pelvic tumor had spread, so the patient underwent radiation therapy. After second-line chemotherapy with doxorubicin, left pleural effusion and mediastinal lymph node metastasis appeared, and third-line chemotherapy with eribulin mesylate was administered. The pleural effusion improved, but the patient developed cough again, and trabectedin was administered as the fourth chemotherapy. In this case, there was no local recurrence for three years after radiotherapy, suggesting the effectiveness of radiotherapy in local control.

## Introduction

Extraskeletal myxoid chondrosarcoma (EMC) of the vulva is extremely rare, with only 14 cases reported in the literature. Sarcomas account for 1-2% of vulvar cancers. EMC is an aggressive sarcoma with a high rate of local recurrence (30-50%) and metastasis (30-50%), although long-term survival is high. Patients with this diagnosis are managed with surgery for the primary tumor, and chemotherapy and radiation therapy have been reported to be less effective [1]. According to previous reports, EMC does not have specific clinical features. It often presents as a single soft tissue lesion that grows slowly month by month and is 5-10 cm in diameter [2]. The lower extremities are a typical site of occurrence, and although they sometimes present with an invasive clinical course, they have shown potential for long-term survival [3]. We describe a case of EMC of the vulva that was controlled with radiation therapy for local recurrence.

## Case presentation

A 41-year-old Japanese woman who reported one pregnancy and one delivery (G1P1C1) became aware of the sensation of a mass in the right vulva, the size of a ping-pong ball with no mobility, in May 2018. She had no history or medical condition that interfered with her daily life, so she remained at home for a year. However, in May 2019, she visited a clinic after noticing an increase in the size of the mass and worsening throbbing pain. Initially, it was thought to be a Bartholin's cyst due to vulvar swelling; however, ultrasound findings showed probable enlargement with multifocal cysts, and the patient was referred to our university hospital and department. Ultrasonographic findings showed elastographic hardness varied from site to site, and Doppler evaluation showed blood flow. A mass was noted in the vulvar region and within the adductor magnus muscle, but it was difficult to measure the largest diameter on this examination.

**Figure 1:**
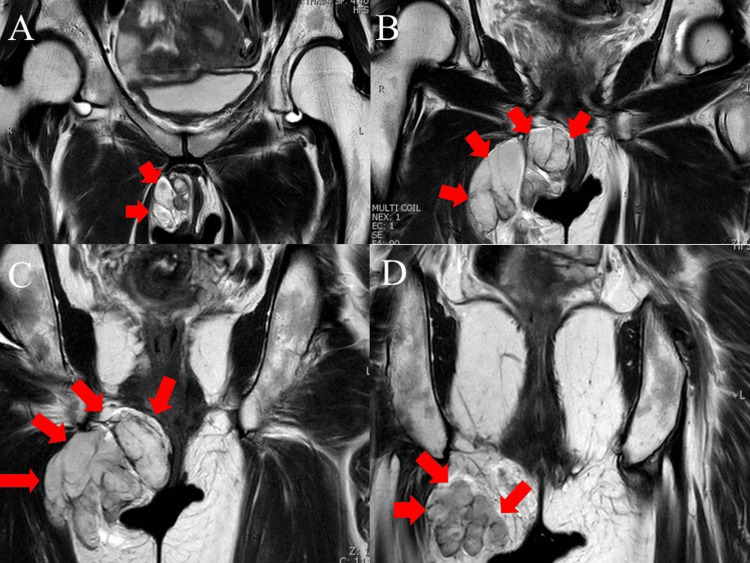
Magnetic resonance images. Coronal section of T2-weighted image. T2-weighted image shows a hyperintense signal. The images from A to D are ventral to dorsal.

**Figure 2:**
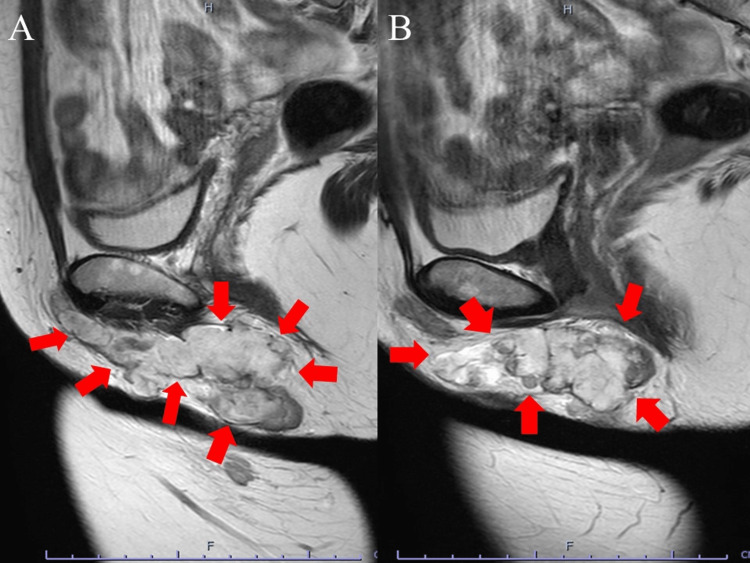
Magnetic resonance images. Sagittal section of T2-weighted image. T2-weighted image shows a hyperintense signal. The image in B is more on the central side than in A.

**Figure 3:**
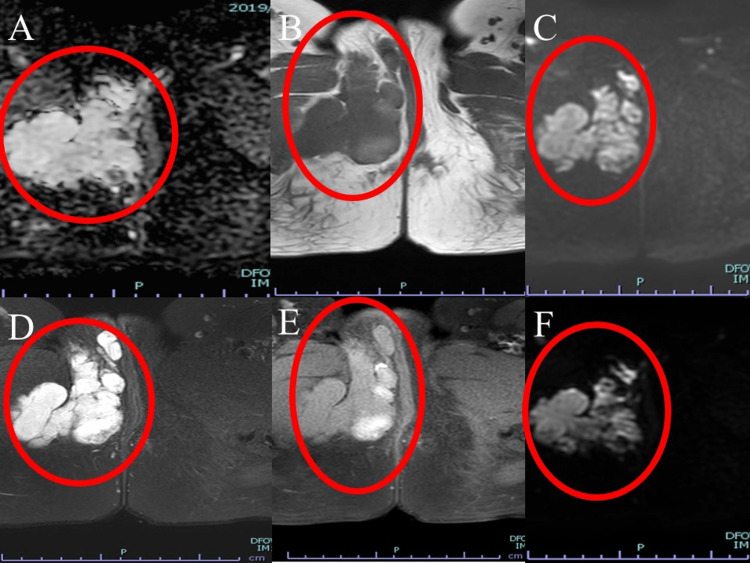
Magnetic resonance images. Various types of axial dissection. The T1-weighted image showed a mild hypointense signal. Diffusion-weighted images (DWI) showed a hyperintense signal, and apparent diffusion coefficient (ADC) maps showed a hypointense signal. A is an ADC image. B is a T1-weighted image. C is a diffusion-weighted image with a b-value of 1000s/mm^2^. D is a fat-suppressed T2-weighted image. E is a fat-suppressed T1-weighted image. F is a diffusion-weighted image with a b-value of 0s/mm^2^.

Physical examination revealed a body mass index of 23.71 kg/m^2^. Magnetic resonance imaging (MRI) showed a tumor between the right perineal skin and the right thigh medial deep muscle, femoral adductor muscle, with hyperintense signal on T2-weighted images (Figures 1-2). It was multifocal and extended into the right thigh adductor muscle. There was no anal or vaginal traffic. T1-weighted imaging showed a mild hypointense signal, suggesting a mucinous tumor. Diffusion-weighted imaging (DWI) showed a hyperintense signal in the perineum and part of the adductor magnus muscle, and apparent diffusion coefficient (ADC) maps showed areas of hypointense signals, findings that were suspicious for malignancy (Figure 3). Sequentially, the mass lesion showed an equal to low signal at T1, a high signal at T2, a high signal on DWI, and a low signal on the ADC map.

A hard, elastic mass lesion approximately 15 cm in size was observed on the medial side of the anorectal aspect of the right thigh, extending to the labia majora and encompassing the buttocks. An internal image was obtained, indicating an internal hemorrhage. No erythema, inflammation, or pulsation was observed. The anterior surface of the pubic bone was elevated, in continuity with the mass. The vaginal Introtus was deviated to the left side due to pressure from the mass, but the mass was not in contact with the vaginal wall on bilateral examination. 

Blood test results revealed no obvious abnormalities. Ultrasonography revealed a multifocal cystic mass in the vulva. In addition, an irregularly shaped, well-defined mass with a heterogeneous internal echo pattern and low echo luminance was observed on the medial thigh. No obvious abnormalities were observed in the uterus or ovaries. A primary mass lesion of the vulva was considered.

**Figure 4:**
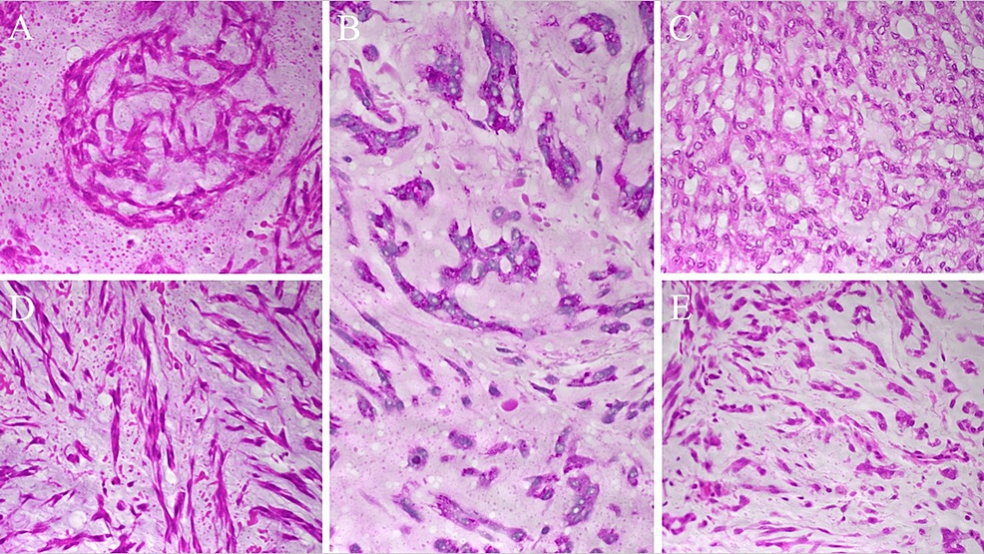
Pathology diagram. Image B in the center showed PAS staining, other images (A, C, D, and E) showed spindle-shaped, polygonal, and round tumor cells with hematoxylin and eosin staining. These cells proliferated in a cordate or reticulate fashion within the matrix of the myxoma. A, tumor cells present a multinodular structure. B, spindle-shaped tumor cells are present in a mucus-like matrix. C, small tumor cells are present in a reticular pattern. D, tumor cells are present in a cord-like structure. E, tumor cells are spindle-shaped, polygonal, or round. All images were taken with an indirect lens at 10x and an objective lens at 40x. (OLYMPUS BX53)

**Figure 5:**
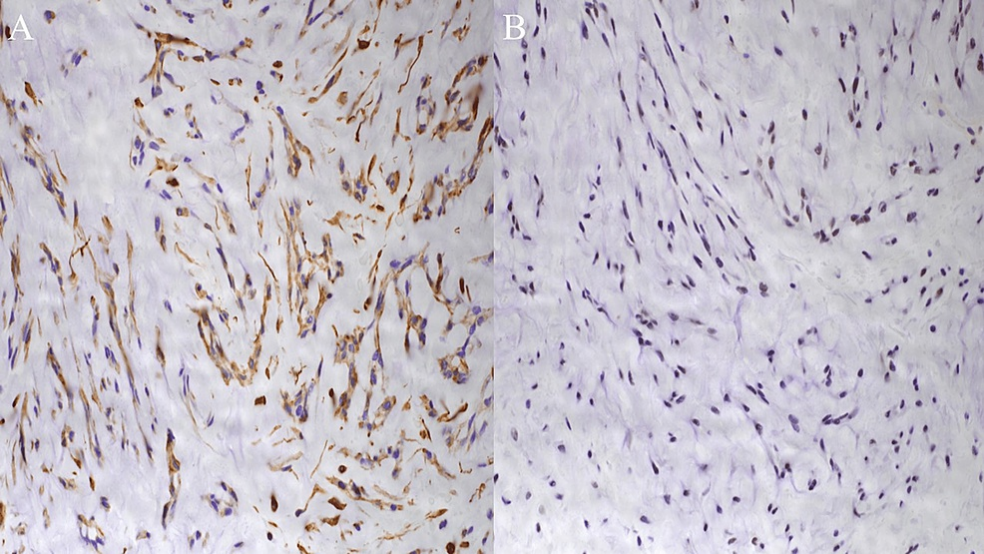
Immunostaining A shows positive for vimentin. B indicates that INI-1 does not become negative.

The patient was examined by an orthopedic surgeon, due to the involvement of the mass within the thigh muscle, and a needle biopsy was performed in August 2019, due to the potential of a malignant soft tissue tumor. Pathologically, as shown in Figures 4-5, hematoxylin and eosin staining revealed spindle, polygonal, and round tumor cells, which appeared mucinous on periodic acid Schiff staining. They proliferated in a cordate or reticulate manner within the myxomatous matrix. In some locations, they proliferated in a nodular manner. Immunostaining results were positive for vimentin and integrase interactor 1 (Figure 5), and negative for cytokeratin AE1/AE3, cytokeratin 7, cytokeratin CAM 5.2, hematopoietic progenitor cell antigen CD34, also called MIC2, T cell surface glycoprotein E2, p30/32 protein (CD99), epithelial membrane antigen (EMA), desmin, α-smooth muscle actin, S100 protein, chromogranin A, and synaptophysin.

EMC was suspected, and additional tests were performed. In the FoundationOne®CDx report (Foundation Medicine Inc., USA), in cancer genome profiling testing, a fusion gene search identified the EWSR1 (ex11)-NR4A3 (CHN,ex3) fusion gene, confirming a diagnosis of EMC. No other significant genetic variants were identified in the present study.

In September 2019, a whole-body positron emission tomography scan revealed no obvious metastases. The tumor was classified as vulvar cancer TNM stage II (FIGO, 2008; NISSANPU, 2014) [4], pT2NXM0. In November 2019, a combined right vulvar and adductor muscle resection was performed as a wide resection of the tumor, and a rectus abdominis valvuloplasty was performed. The final postoperative diagnosis was right adductor muscle, pubococcygeal periosteum, and pubic mound skin invasion with a primary right vulvar EMC.

In January 2020, postoperative adjuvant radiation therapy (external-beam radiation, 60 Gy/30 fractions) was administered from the pelvis to the vulva and right thigh, and in July 2020, pazopanib was initiated as first-line chemotherapy. Lung metastasis was controlled, but the pelvic tumor increased (38x51x46mm at MRI), and radiation therapy (external-beam radiation, 60 Gy/3 fractions) was administered in April 2021; in October 2021, dyspnea and cough appeared, and computed tomography showed left pleural effusion and mediastinal lymph node metastasis. The progression-free survival after second-line chemotherapy was seven months. After tertiary chemotherapy with eribulin mesylate, the pleural effusion improved, but the patient developed a cough due to enlarged mediastinal lymph nodes. Trabectedin was given as 4th line treatment for mediastinal lymph node metastasis.

## Discussion

Malignant soft tissue tumors are rare, with an estimated incidence of 3.6 per 100,000 [5]. There are more than 30 subtypes of soft tissue sarcomas, with liposarcoma, anaplastic polymorphous sarcoma, and leiomyosarcoma being the most prevalent [5]. Soft tissue sarcomas are generally more common in men, whereas leiomyosarcomas and synovial sarcomas are more common in women [5]. According to the histological classification, extraosseous chondrosarcoma is classified as a cartilage and osteogenic tumor. The subcategories are highly differentiated, myxoid, mesenchymal, and dedifferentiated tumors. These tumors account for 2.5-3% of all soft tissue sarcomas and are characterized by multinodular structures, mucous-like matrices, and malignant chondroblasts [6]. However, the histogenesis of EMC remains controversial, and EMC is currently classified as a mesenchymal tumor of uncertain differentiation [7].

EMC is characterized by a fusion gene, Ewing sarcoma (EWS) RNA-binding protein 1-nuclear receptor subfamily 4, group A, member 3 (EWSR1-NR4A3), which is useful for differential diagnosis [8,9]. However, EMC of the vulva is extremely rare, with only 14 cases reported in the literature. EMC is poorly characterized on MRI, as most myxoid tumors are characterized by soft tissue masses that show hyperintense signals on T2-weighted images [3]. Pathologically, EMC is considered a tumor of uncertain differentiation and is difficult to differentiate because the cartilage matrix is generally not observed. However, previous studies have reported the presence of gene fusions specific to EMC, namely chromosomal reciprocal translocation t(9;22)(q22;q12) [10], and EWSR1/NR4A3 gene fusion is the most common genetic event in EMC, occurring in 75% of the classical forms [11].

In our case, the EWSR1 (ex11)-NR4A3 (CHN, ex3) fusion gene was confirmed using a cancer genome profiling test, FoundationOne®CDx (F1CDx, Chugai Pharma Co. Japan; Foundation Medicine, Inc., USA), in addition to MRI and histopathology, thereby confirming the diagnosis. The genetic diagnosis of sarcomas is becoming mainstream.

Regarding treatment, multivariate analysis of all EMCs reported to date has shown that chemotherapy (hazard ratio (HR), 6.054; 95% confidence interval (CI), 1.33-27.7; p = 0.020) and radiation therapy (HR, 5.07; 95% CI, 1.3-20.1; p = 0.021) were effective expectations are considered low [12]. In our case, there was no local recurrence after radiotherapy, suggesting the efficacy of radiotherapy for local control. As periosteal invasion was suspected at the time of surgery, radiotherapy was administered as adjuvant therapy, and the lesion was controlled within the irradiated area after the initial irradiation. Seven months after the surgery, radiotherapy was administered for pelvic recurrence in the common iliac lymph node within the irradiated area, and the disease was successfully controlled. Distant metastatic lesions such as multiple lung metastases were diagnosed using F1CDx, and pazopanib was used as the primary chemotherapy according to previous reports. Data on the efficacy of drug therapy for metastatic EMCs in previous reports are limited, but anthracycline regimens, trabectedin, sunitinib, and pazopanib have shown some efficacy [12]. In the present case, drug therapy appeared to have controlled the pulmonary metastases; pazopanib was successful and resulted in progression-free survival of 15 months. Local control can be achieved with radiation therapy; however, chemotherapy is the only option for conditions that are difficult to treat with radiation therapy, such as mediastinal lymph node enlargement. A long-term follow-up is necessary to confirm the efficacy of this therapy.

## Conclusions

Vulvar EMC is rare. As radiation is adequate for local metastases and possibly for distant metastases, there's a role in the postoperative management schedule. For radical surgery, our patient underwent joint surgery with orthopedic surgery in a team medicine setting. For a definitive diagnosis, cancer tumor molecular profiling was useful in addition to histopathology. Localized radiation therapy was especially effective as adjuvant therapy. There was a recurrence of pelvic lesions outside the initial irradiation field, but it was controlled by radiation therapy. Genetic diagnosis is becoming the mainstay in sarcomas of unknown differentiation. Chemotherapy possibly has an additional role in distant metastases. Pazopanib was effective as initial chemotherapy in this case. This case demonstrated the treatment necessary for EMC control in the future. Radiation therapy may be an effective adjuvant therapy.
